# Management of acute coronary syndromes requiring coronary angiography in patients with drug reaction with eosinophilia and systemic symptoms syndrome induced by iodinated contrast media: two case reports and narrative review

**DOI:** 10.1093/ehjcr/ytae259

**Published:** 2024-05-21

**Authors:** Carola Griffith Brookles, Matteo Bianco, Stefano Pizzimenti, Giulia Gobello, Carloalberto Biolè, Paola Destefanis, Enrico Cerrato, Alessandra Chinaglia

**Affiliations:** Cardiology Division, San Luigi Gonzaga University Hospital, Regione Gonzole 10, 10043 Orbassano, Turin, Italy; Department of Medical Sciences, University of Turin, Turin, Italy; Cardiology Division, San Luigi Gonzaga University Hospital, Regione Gonzole 10, 10043 Orbassano, Turin, Italy; Severe Asthma and Rare Lung Disease Unit, San Luigi Gonzaga University Hospital, Turin, Italy; Cardiology Division, San Luigi Gonzaga University Hospital, Regione Gonzole 10, 10043 Orbassano, Turin, Italy; Department of Medical Sciences, University of Turin, Turin, Italy; Cardiology Division, San Luigi Gonzaga University Hospital, Regione Gonzole 10, 10043 Orbassano, Turin, Italy; Cardiology Division, San Luigi Gonzaga University Hospital, Regione Gonzole 10, 10043 Orbassano, Turin, Italy; Cardiology Division, San Luigi Gonzaga University Hospital, Regione Gonzole 10, 10043 Orbassano, Turin, Italy; Cardiology Division, San Luigi Gonzaga University Hospital, Regione Gonzole 10, 10043 Orbassano, Turin, Italy

**Keywords:** DRESS syndrome, Allergic reaction prevention, Iodinated contrast media, Coronary angiography, Premedication, Case report

## Abstract

**Background:**

Hypersensitivity reactions to iodinated contrast media (ICM) are frequently encountered in clinical practice. Severe manifestations, despite being infrequent, can be life-threatening and represent an issue when re-administration of ICM is required. Clear recommendations on prevention and management of relapses are still lacking.

**Case summary:**

We present the cases of two patients presenting with acute coronary syndrome requiring urgent coronary angiography, with an anamnesis of ICM-induced drug reaction with eosinophilia and systemic symptoms syndrome. Both patients safely underwent a coronary angiography with the use of a different ICM (iobitridol) to the one linked to hypersensitivity manifestations, after premedication with corticosteroids and H1 antagonists.

**Discussion:**

Our experience highlights that in clinical situations in which the use of ICM is urgently needed, premedication with corticosteroids and H1 antagonists together with the choice of an alternative contrast agent (when the culprit is known) represents an effective strategy to perform a potentially life-saving procedure while avoiding serious systemic allergic reactions.

**Learning points**

1. Hypersensitivity reactions to iodinated contrast media (ICM) are frequently encountered in clinical practice.
2. Severe manifestations like drug reaction with eosinophilia and systemic symptoms syndrome, despite being infrequent, can be life-threatening.
3. Clear recommendations on prevention and management of relapses are still lacking.
4. In case of acute coronary syndrome, when ICM for coronary angiography is urgently needed, premedication with corticosteroids and H1 antagonists together with the choice of an alternative contrast agent (when the culprit is known) could represent a safe and effective strategy.

## Introduction

Immediate and non-immediate allergic reactions to iodinated contrast media (ICM) are frequently encountered in clinical practice and are classified according to the time interval between the exposure to the contrast media and the development of clinical manifestations. Even though they are majorly represented by self-limited cutaneous reactions, few patients do develop more severe and rare manifestations that require monitoring in a protected environment and specific treatment.^1^

It is not uncommon for patients with a history of allergic reactions to contrast media to require radiologic studies with the use of ICM.

In these cases, it is essential to balance between the risk deriving from the re-administration of a known allergen, especially when previous manifestations have been severe, and the urgency to undergo a radiologic study or procedure. Furthermore, there are no clear recommendations on the optimal strategy to prevent their re-occurrences.

Here, we present the cases of two patients with a history of ICM-related drug reaction with eosinophilia and systemic symptoms (DRESS) syndrome, one of the most severe non-immediate manifestations in the spectrum of allergic reactions, who presented with acute coronary syndrome, thus requiring urgent coronary angiography.

## Summary figure

Summary figure: the figure summarizes the management of patients with previous severe delayed allergic reaction to ICM.

**Figure:**
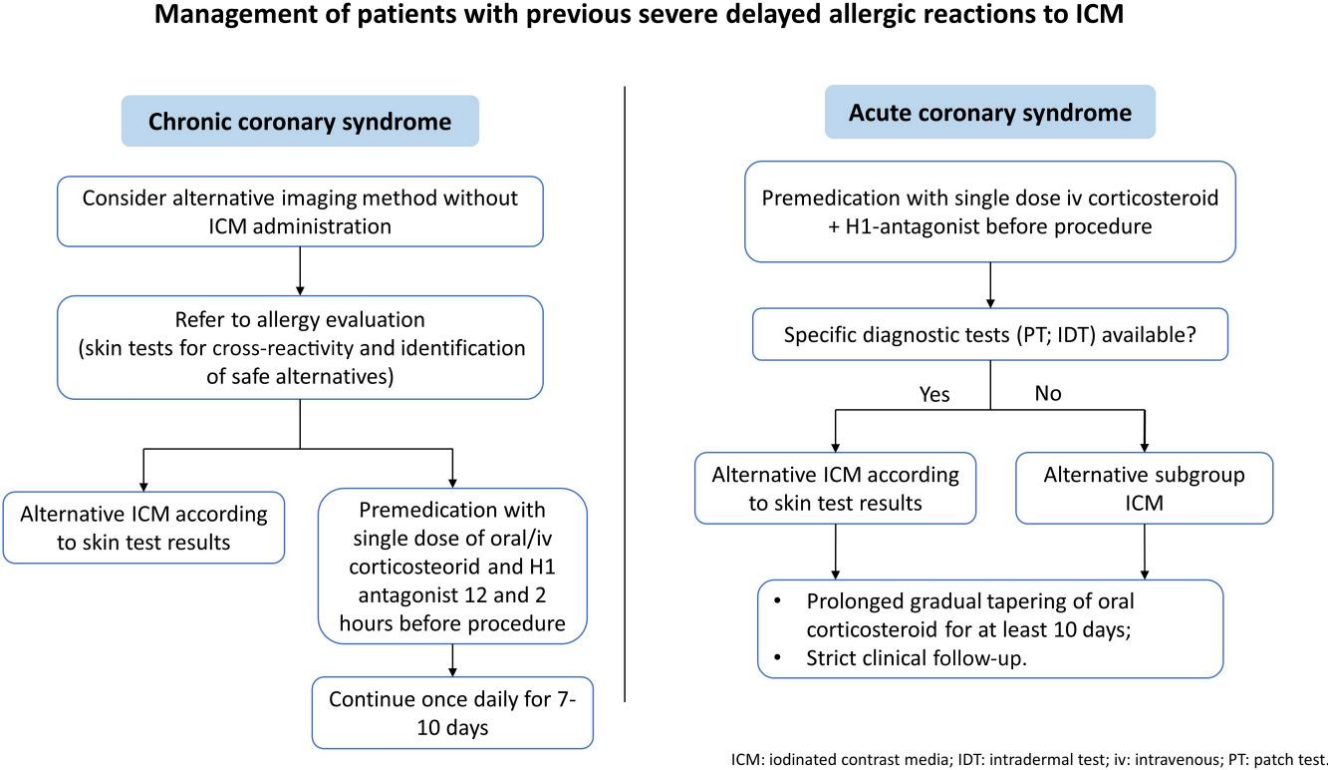


## Clinical cases

### Case 1

**Figure 1:**
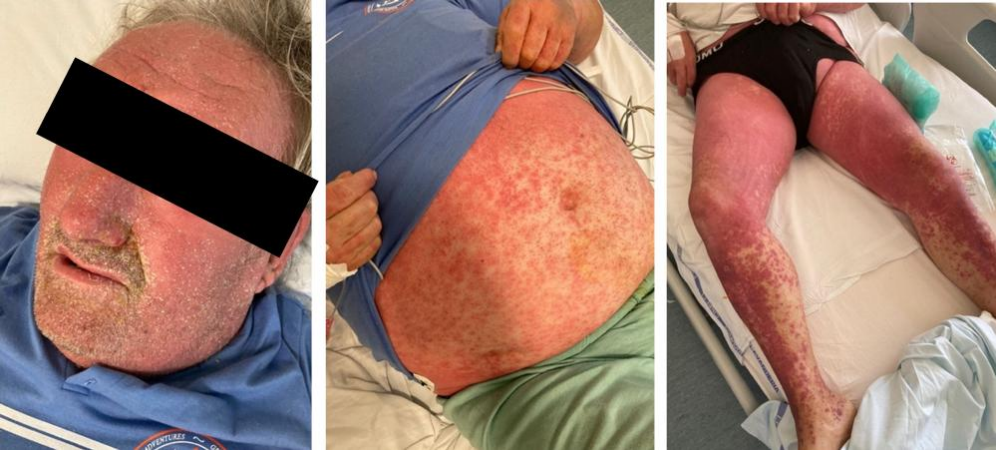
Erythroderma and cutaneous purpura, configuring cutaneous manifestations of drug reaction with eosinophilia and systemic symptoms syndrome.

A.B. is a 69-year-old diabetic man who presented to the emergency department (ED) for precordial chest pain persisting for 7 days associated with worsening dyspnoea. Due to his respiratory symptoms, he had been using self-managed high doses of salbutamol. He had a history of three-vessel coronary artery disease and had already undergone multiple percutaneous revascularizations. After the last coronary angiography, he had developed a severe systemic allergic reaction to the ICM (using iomeprol, brand name Iomeron, Bracco), namely DRESS syndrome, which presented as erythroderma, cutaneous purpura, cholestasis, and eosinophilia and for which he had been treated with high-dose systemic corticosteroids (*Figure 1*). As relevant comorbidities, he had permanent atrial fibrillation, immunosuppressor-dependent microscopic polyangiitis, chronic kidney disease, and a type II bipolar disorder. His medications included metformin (1000 mg t.i.d.), apixaban (2.5 mg b.i.d.), aspirin (100 mg m.i.d.), metoprolol (100 mg b.i.d.), potassium canrenoate (50 mg m.i.d.), and furosemide (25 mg b.i.d).

Upon arrival, he was tachycardic but haemodynamically stable. The electrocardiogram showed high-penetrance atrial fibrillation and signs of anterior necrosis (V2–V4) with a slight ST elevation (maximum 0.5 mm) in the same leads. The laboratory tests revealed at-plateau increase in high-sensitivity cardiac troponins (hs-cTnI; first determination, 236 pg/mL; second determination, 229 pg/mL) and elevated inflammatory markers (C-reactive protein 13.25 mg/L). The transthoracic echocardiogram showed severe, circumferential, partially organized pericardial effusion without haemodynamic impact, apical akinesia, medial segments’ hypokinesia with a severely reduced ejection fraction (30%) and mild mitral regurgitation. In the suspect of a late referral acute coronary syndrome, he was admitted to the intensive care coronary unit. During the following days, he developed pleuritic pain. In the suspect of Dressler’s pericarditis, therapy with non steroidal antiinflammatory drugs and colchicine was started, with a subsequent gradual improvement of symptoms and progressive reduction of pericardial effusion.

**Figure 2:**
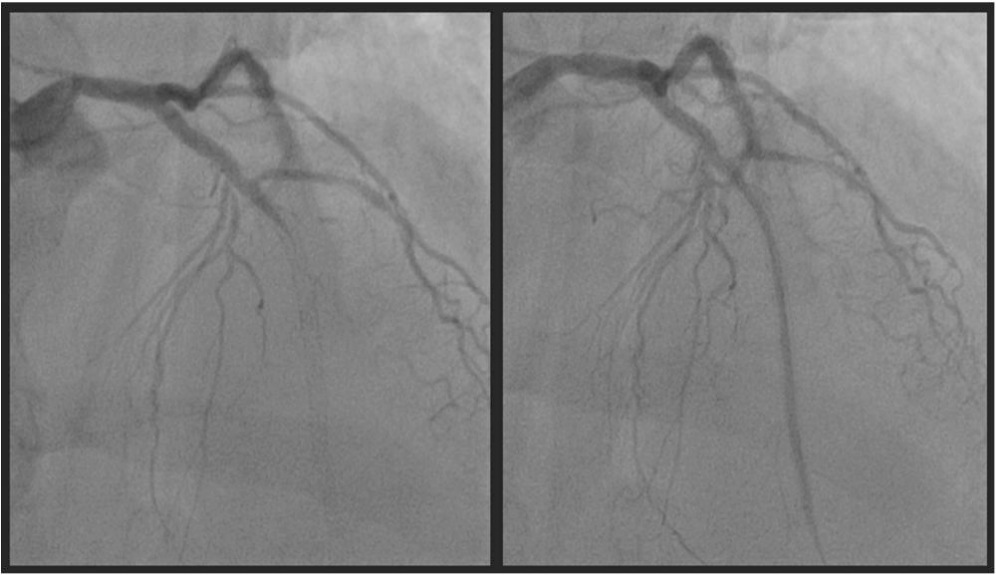
Coronary angiography with iobitridol as contrast medium, showing a complete intra-stent occlusion of the distal tract of the descending anterior coronary artery, treated with balloon angioplasty.

**Figure 3:**
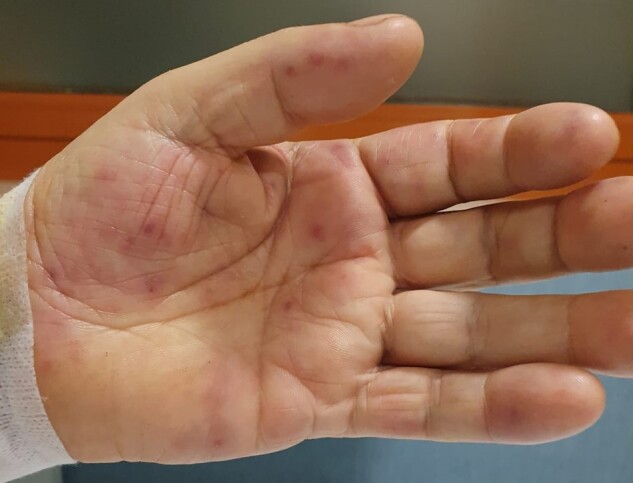
Pruritic purpuric lesions developing after the coronary angiography.

During hospitalization, he presented recurrent episodes of non-sustained ventricular tachycardia and relapses of anginal chest pain. Therefore, it was decided to perform a coronary angiography. Considering the development of contrast media–induced DRESS syndrome after the administration of iomeprol (Iomeron, Bracco, Subgroup A) during a previous hospitalization, an allergologist evaluation was requested before the procedure. Due to the urgent clinical situation, it was impossible to stratify the risk of a new systemic allergic reaction and assess tolerability to other ICMs through skin tests. Premedication with high-dose methylprednisolone and cetirizine was started, and the use of an alternative subgroup ICM, iobitridol (Xenetix, Guebert, Subgroup B), was proposed. Coronary angiography (with the use of iobitridol as ICM) then showed a complete intra-stent occlusion of the distal tract of the descending anterior coronary artery, successfully treated with balloon angioplasty (*Figure 2*). After the procedure, the patient prosecuted first intravenous and then oral corticosteroid therapy, gradually tapering doses for 3 weeks. In the days following ICM administration, he developed pruritic purpuric lesions on both palmar regions. Upon allergy consultation, any relation to an allergic reaction to the contrast media was excluded, as the purpuric lesions were correlated to the patient’s chronic comorbidities (micropolyangiitis), and he was advised to undergo re-evaluation at his reference centre after discharge (*Figure 3*).

### Case 2

M.G. is a 66-year-old diabetic man who presented to the ED for relapsing chest pain. The previous day, he had self-discharged from another hospital with a diagnosis of non-ST-elevation myocardial infarction after refusing to undergo a coronary angiography. He had a history of three-vessel coronary artery disease treated with multiple percutaneous revascularizations. After one of these procedures (using iohexol as ICM), he had developed DRESS syndrome, presenting with fever, diffuse cutaneous rash, increase in transaminases and inflammatory markers, and moderate eosinophilia, which had been successfully treated with high-dose corticosteroids. Over the course of the following 3 months, and almost 6 months before presentation to our cardiology division, he had undergone prick tests and intradermal tests (IDTs) to confirm susceptibility to iohexol and assess cross-reactivity with other ICMs. The results showed a negative prick test and a positive IDT at 48 h to iohexol, while iopromide, iobitridol, and gadoteric acid tested negative for cross-reactivity.

As relevant comorbidities, he had recently received a diagnosis of pleural mesothelioma with an indication to start chemotherapy with pemetrexed/carboplatin. His medications included insulin, aspirin (100 mg m.i.d.), metoprolol (50 mg b.i.d.), and ramipril (2.5 mg m.i.d.).

On admission, he was haemodynamically stable and asymptomatic. His vital parameters were within the normal range. The electrocardiogram showed sinus rhythm (heart rate 96 b.p.m.) and normal atrioventricular and intraventricular conduction with flat/negative T waves in DIII-aVL. The laboratory tests showed a decrease in hs-cTn (on the previous day: hs-cTnI, first determination 55 pg/mL and second determination 558 pg/mL; hs-cTnI on admission, 424 pg/mL) and mild anaemia. Considering the diagnosis of acute coronary syndrome, he was admitted to the intensive care coronary unit, and a coronary angiography was indicated. An allergologist was consulted: given the results of previous skin tests, the use of iobitridol as ICM was suggested, and premedication with methylprednisolone and cetirizine was indicated. Coronary angiography (with the use of iobitridol as ICM) then showed a significant stenosis of the posterolateral branch of the circumflex coronary artery, treated with angioplasty and stent insertion, and an intra-stent restenosis of the medial tract of the anterior descending coronary artery, successfully treated with balloon angioplasty. Treatment with intravenous and subsequently oral corticosteroids and oral cetirizine was continued, gradually tapering doses in the following 10 days.

During the days following the procedure, both patients did not display any sign or symptom suggestive of a relapse of DRESS syndrome, while levels of eosinophils, transaminases, and cholestasis indices persisted within the normal range.

## Discussion

Drug reaction with eosinophilia and systemic symptoms syndrome is a non-immediate drug reaction that usually presents with an extensive skin rash, fever, haematologic abnormalities such as systemic eosinophilia and atypical lymphocytosis, and involvement of at least one organ (i.e. hepatitis) and has been linked to herpesvirus reactivation. Involvement of internal organs can be severe and difficult to recognize. Together with the frequent autoimmune and virologic sequelae, it justifies the elevated mortality (up to 10%) associated with the syndrome.^2,3^

Even though DRESS syndrome is most frequently associated with the administration of anticonvulsants, cases related to ICM have been described and can manifest shortly after exposure.^4,5^ Patients with cardiovascular diseases (especially when treated with beta-blockers) or oncologic comorbidities are at higher risk of developing hypersensitivity reactions to ICM.

When handling a patient with a hypersensitivity reaction, the first therapeutic measure is the identification and suspension of the causing agent or medicament. In this fashion, avoiding re-exposure to the known allergen is the first way to prevent re-occurrences of severe hypersensitivity manifestations that can be lethal. However, especially in cases of allergic reaction to ICM, it is not infrequent for patients to undergo other radiologic procedure requiring their administration. Furthermore, contrast media have demonstrated a significant cross-reactivity in eliciting allergic reactions,^6^ especially for those with structural similarities. Cross-reactivity does not appear to be related neither to iodine and ionicity nor to excipients, rather to the presence of carbamoyl side chains in some ICMs; coherently, with these observations, a classification of ICMs in three classes (A, B, and C) based on the presence of the carbamoyl chain and the molecular structure of the ICM was created. It has been proposed that the probability of cross-reactivity is lower when considering inter-class ICMs, while being elevated for ICMs belonging to the same group and with structural analogies.^7^ Accordingly, in order to avoid new episodes of DRESS syndrome, the two presented cases, who had been initially treated with iohexol and iomeprol, both Class A ICMs, successfully received iobitridol—a Class B ICMs, as contrast media for their new coronary angiography.

Therefore, ensuring the possibility of undergoing a necessary procedure while avoiding severe reactions appears challenging but feasible.

After the development of a severe delayed drug allergic reaction, international guidelines recommend a series of diagnostic tests to confirm the causative agent and assess cross-reactivity, which include patch tests (PTs) and IDTs.^1,8^ However, it is frequent to encounter patients who, despite an anamnesis of allergic reactions to ICM, did not undergo specific diagnostic tests before requiring re-administration. When there is a clinical urgency, it is impossible to postpone the procedure to conduct examinations that would better stratify the risk of recurrence of hypersensitivity manifestations and help identify an alternative ICM with good tolerability. Therefore, management of these patients is challenging.

Premedication with corticosteroids and H1 antihistamines is frequently used when managing allergic reactions. However, it should be borne in mind that while its role has been validated for specific chemotherapeutic protocols, its efficacy has not been demonstrated in case of ICM-induced allergic reactions and repeated reactions have been reported despite premedication.^8^

Available guidelines suggest against routine use of premedication with corticosteroids and H1 antihistamines prior to re-administration of ICM in patients with anamnesis of allergic reactions. The rationale for this is the lack of evidence showing a convincing benefit and the risk of breakthrough reactions in patients with previous severe reactions despite premedication administration.^8^ However, they do recognize a potential added value in case of severe and delayed manifestations.^9^

In contrast with the uncertainty of recommendations, premedication is still widely diffused in clinical practice. Depending on the urgency of clinical situation, corticosteroids can be administered orally or intravenously. The most diffuse scheme involves three doses of oral corticosteroid in the 24 h preceding the procedure, followed by a rapid injection of H1 antagonist before it starts. This pharmacologic therapy prevents and contrasts the effects of basophil activations, which is directly elicited by ICM in immediate hypersensitivity reactions.^10^

Conversely, in patients with a history of potential drug delayed reactions, the most diffuse strategy is a single dose of oral or intravenous corticosteroid and H1 antagonist 12 and 2 h before the procedure. Corticosteroid therapy is usually continued once daily after the procedure for at least 7–10 days.

The use of an alternative ICM has demonstrated to reduce the risk of re-occurrence of hypersensitivity reactions; nevertheless, assessing cross-reactivity and good tolerability of alternatives through skin tests remains the optimal strategy to identify a safer ICM.^11^ Unfortunately, skin tests maintain a good predictive value only within 2–6 months after the suspected reaction, probably due to IgE rapid clearance. However, DRESS represents a T-cell hypersensitivity delayed reaction: a drug-specific immune response that can be proven with the use of PTs or IDTs, as well as the *in vitro* demonstration of drug-specific CD4+ and CD8+ T cells producing large amounts of tumour necrosis factor-alpha and interferon-gamma.^12,13^

In the two presented cases, the urgency of the clinical situation required rapid administration of ICM, according to the recommendations on the management of acute coronary syndromes.^14^ Both patients had displayed an extremely severe allergic reaction to ICM. In the first case, the patient had not undergone further testing: the choice of an alternative contrast media was dictated by evidence regarding the potential tolerability of an alternative subgroup ICM, without the possibility to confirm the absence of cross-reactivity through skin tests. On the other hand, in the second scenario, previous testing indicated three potential alternative ICMs to be used in case of clinical necessity.

Taking into considerations all available evidence, it was decided to combine the use of premedication with corticosteroid and H1 antihistamines with the choice of an alternative ICM, guided by results of previous skin tests when available. This strategy, combined with a prolonged and gradual tapering of oral corticosteroids in at least 10 days and strict clinical follow-up, proved to be a well-tolerated, safe, and effective strategy to ensure the feasibility of a life-saving procedure while avoiding systemic hypersensitivity delayed manifestation. In both our cases, iobitridol was chosen as the alternative ICM, and its administration did not result in relapses of hypersensitivity manifestations and was well tolerated, even in the absence of a previous cross-reactivity assessment. Furthermore, its use did not imply organizational issues, as the contrast media was simple to acquire and ensured good-quality angiographic images.

Our approach to ICM adverse reaction is summarized in the *Summary figure*.

## Conclusion

Allergic reactions to ICM still represent a challenge in clinical practice, especially when a medical urgency requires their use in patients at high risk of developing a severe hypersensitivity reaction.

Our experience highlights that in clinical situations in which the use of ICM is urgently needed, premedication with corticosteroids and H1 antihistamines together with the choice of an alternative subgroup contrast agent (when the culprit is known) represents an effective strategy to perform a potentially life-saving procedure while avoiding dangerous systemic hypersensitivity reactions.

Considering frequent cross-reactivity, administration of an alternative ICM in patients with a history of allergic reactions must be followed by a close clinical monitoring, and every sign or symptom that could suggest a relapse of previous hypersensitivity reactions must be addressed carefully, with the support of an allergy specialist.

## Data Availability

The data underlying this article are available in the article and in its online supplementary material. Additional data could be provided upon reasonable requests.

## Lead author biography

Dr Matteo Bianco is a cardiology consultant at San Luigi Gonzaga University Hospital, Orbassano, Italy. He completed Cardiology Specialization in Turin University and a Master in Cardiology Intensive Care in Padua University and a Master in Cardiac Magnetic Resonance in Monzino Hospital, Milan. His main field of interest and research are pharmacological treatment of cardiovascular disease, acute and chronic heart failure, and cardiomyopathies.

**Consent:** The authors confirm that a written consent for the submission and publication of this case report, including images and associated text, has been obtained from the patients in line with the COPE guidelines.

**Funding:** None declared.
